# Evaluating the performance of the Pain Interference Index and the Short Form McGill Pain Questionnaire among Chilean injured working adults

**DOI:** 10.1371/journal.pone.0268672

**Published:** 2022-05-19

**Authors:** Juan Carlos Velez, Lauren E. Friedman, Clarita Barbosa, Jessica Castillo, Diana L. Juvinao-Quintero, Michelle A. Williams, Bizu Gelaye

**Affiliations:** 1 Departamento de Rehabilitación, Hospital del Trabajador, Santiago, Chile; 2 Department of Epidemiology, Harvard T.H. Chan School of Public Health, Boston, MA, United States of America; 3 Multidisciplinary International Research Training (MIRT) Program, Harvard T.H. Chan School of Public Health, Boston, MA, United States of America; 4 The Chester M. Pierce, MD Division of Global Psychiatry, Massachusetts General Hospital; Boston, MA, United States of America; 5 Department of Psychiatry, Harvard Medical School, Boston, MA, United States of America; Mugla Sitki Kocman Universitesi, TURKEY

## Abstract

**Background:**

Chronic pain can lead to economic instability, decreased job productivity, and poor mental health. Therefore, reliable identification and quantification of chronic pain is important for clinical diagnosis and treatment.

**Objective:**

To determine the psychometric properties of the Spanish language versions of the Pain Interference Index (PII) and the Short Form McGill Pain Questionnaire (SF-MPG) among a population of working adults who experienced injury in Santiago, Chile.

**Methods:**

A total of 1,975 participants with work-related injuries were interviewed to collect sociodemographic, occupational, and chronic pain characteristics. Construct validity and factorial structure of the PII and SF-MPG were assessed through exploratory factor analyses (EFA). Cronbach’s alpha was used to evaluate internal consistency.

**Results:**

The PII mean score was 3.84 ± 1.43 among all participants. The SF-MPG median score was 11 [IQR: 6–16] in this study population. Cronbach’s alpha for the PII was 0.90 and 0.87 for the SF-MP. EFA resulted in a one factor solution for the PII. A two-factor solution was found for the SF-MPG. The two-factors for SF-MPG were sensory and affective subscales with Cronbach’s alpha of 0.82 and 0.714, respectively. When the two scales were combined, an EFA analysis confirmed the PII and SF-MPG measure different aspects of chronic pain.

**Conclusions:**

The PII and SF-MPG had good construct validity and reliability for assessing different aspects of chronic pain among working Chilean adults.

## Introduction

Chronic pain is typically defined as pain that persists for longer than the expected time frame for healing (3 to 6 months), and lacking the acute physiological signal of warning (nociception) of acute pain [1]. In a study conducted in 17 countries, investigators estimated a 12-month prevalence of chronic pain conditions at 41.1% in low and middle-income countries and 37.3% in high-income countries surveyed [2]. Additionally, a recent World Health Organization study across 14 countries found that among patients with a persistent pain condition at baseline, 49% had not recovered 12 months later [3]. Chronic pain can be classified into three main categories: nociplastic, neuropathic, and nociceptive [4]. Nociceptive pain is the most common form of chronic pain and includes arthritis and most forms of spinal pain [4]. Low back pain is one of the leading causes of years lost to disability with major socioeconomic costs attributed to work absenteeism [5]. A systematic review of chronic lower back pain in Latin American countries found pain prevalence estimates between 4.2–10.1% [6]. In Argentina, back pain was the third most frequent work-related injury, with an incidence rate of 5.2/1000 worker-years [7]. Furthermore, a recent telephone-based survey shows the estimated prevalence of chronic non-cancer pain was 32.1% of the general population in Chile [8].

Beyond the economic burden, chronic pain is a pervasive global public health problem associated with decreased psychological and physical health. Of note, chronic pain is associated with limitations on daily activities, reduced quality of life, and poor mental health [3, 9, 10]. Chronic pain impacts different domains of life including work, social, recreational and self-care activities [11]. Among working adults, chronic pain has large effects on the workforce and economic productivity. In a recent World Health Organization (WHO) World Mental Health survey, pain conditions accounted for 21.5% of all days per year that workers were unable to work or carry out normal daily activities [9]. Using the 2019 National Health Interview Survey (NHIS), Yong *et al*. found half of U.S. adults (50.2%) reported experiencing chronic pain and significantly more days of work missed compared to those without pain (10.3 vs 2.8 days) [12]. A recent study of the Chilean health system found 31.8% and 27.1% of annual expected health system costs were due to lower back pain and osteoarthritis, respectively [13]. As a result, consistent and reliable identification of chronic pain is important for clinical diagnosis, treatment, and long-term follow-up. Reliable methods of quantification for chronic pain are needed for further research and treatment of pain conditions. The Pain Interference Index (PII) was developed in 2009 to measure the extent to which pain has interfered with daily activities in the past two weeks [14]. The PII was originally developed for pediatric populations but has since been used in studies of adolescents and adults with chronic pain [14–16].

Another widely used scale is the McGill Pain Questionnaire which was developed as a multidimensional measure of perceived pain among adults with chronic pain [17]. The McGill Pain Questionnaire was introduced in 1975 and includes 78 words related to sensory, affective, evaluative, and miscellaneous pain subscales [17]. The McGill Pain Questionnaire was the first measure of multiple dimensions of pain; previous scales had focused only on pain intensity. The Short Form McGill Pain Questionnaire (SF-MPG) was subsequently developed in 1987 and includes pain rating items related to sensory and affective subscales of pain [18].

Despite their increased utility in clinical and population-based studies, to the best of our knowledge, no previous study has validated the PII or SF-MPG in Spanish-speaking populations. Therefore, the objective of our study was to determine the psychometric properties of the (1) Pain Interference Index (PII) and (2) Short Form McGill Pain Questionnaire among a population of working adults who experienced an injury in Santiago, Chile.

## Methods

### Study population

Study participants were from the Stress, Pain, Sleep, and Neuropsychiatric Disorders (SPLENDID) Study in Santiago, Chile. The SPLENDID study was conducted between September 2015 and February 2018. The overarching goals of the SPLENDID study were to examine pain, work-related stress, and neuropsychiatric outcomes among working adults attending the Hospital del Trabajador in Santiago, Chile with an intention of designing workplace intervention programs. The Hospital del Trabajador is the biggest workers’ compensation hospital and the referral center for trauma and professional diseases of Asociación Chilena de Seguridad, the largest workers’ compensation system in Chile, with more than two and a half million affiliated workers. Using convenience sampling, participants were approached and interviewed during their outpatient visit to one of the rehabilitation clinics at the hospital. Participants were eligible for the study if they were able to read and write Spanish and attended the Hospital del Trabajador outpatient departments for the following types of injuries: spinal cord, mild brain injury, bone fractures, burns, and soft tissue injuries of various etiologies. Participants were excluded if they did not read or write Spanish.

### Data collection

After informed consent, each participant was interviewed face-to-face by a trained interviewer using a structured questionnaire. The questionnaire ascertained demographic information including age, sex, and education level. Questions were also included regarding participants’ occupation and their pain symptoms. The questionnaire was originally written in English, translated into Spanish by a team of native Spanish speakers with experience in pain research. To ensure proper expression and conceptualization of terminologies in local contexts, the translated version was back-translated and modified until the back-translated version was comparable with the original English version. This was important to ensure that the translated questionnaires are conceptually identical to the English versions. All participants provided written informed consent. The institutional review boards of the Hospital del Trabajador, Santiago, Chile, and the Office of Human Research Administration, Harvard T.H. Chan School of Public Health, Boston, MA approved all study procedures and research protocols. Additional information regarding the ethical, cultural, and scientific considerations specific to inclusivity in global research is included in the Supporting Information (S1 Checklist of inclusivity in global research).

### Analytic population

A total of 2,000 participants were interviewed for the SPLENDID study. For the purpose of our analysis, 3 participants were excluded for missing information on the PII, 21 participants were excluded for missing information on the SF-MPG and 1 participant was excluded for being outside the study age range [18–85]. A total of 1,975 participants were included in the final analysis.

### Pain Interference Index

The Pain Interference Index (PII) assesses the extent to which pain has interfered with daily activities in the past two weeks prior to assessment [15, 16]. PII contains 6 items on a 7-point Likert scale ranging from 0 (not at all) through 6 (complete). Mean PII score ranges from 0–6, with higher scores indicating pain is more likely to interfere with daily activities.

### Short-Form McGill Pain Questionnaire

The Short-Form McGill Pain Questionnaire (SF-MPG) is a multidimensional measure of current perceived pain among adults with chronic pain [18]. The main component of the SF-MPG is a 15-item questionnaire comprised of 2 subscales: 1) a sensory subscale with 11 items and 2) an affective subscale with 4 items. Each item is rated on a Likert intensity scale with 0 = none, 1 = mild, 2 = moderate, or 3 = severe. The total SF-MPG score was obtained by summing the item scores (range 0–45). The sensory subscale ranges from 0–33, and the affective subscale ranges from 0–12 [18–20].

### Covariates

Participants’ age was categorized as 18–24, 25–34, 35–44, 45–54, 55–64, 65–74, and 75–84 years. Additional sociodemographic characteristics examined were sex (male vs. female), country of birth (Chile vs. others), belonging to indigenous or native groups (no vs. yes), highest degree of education attained (elementary school, high school, college or technical training), marital status (married/living with a partner, single, or previously married). Height and weight were measured with participants wearing light clothing and no shoes. Body mass index (BMI) was calculated as weight (kg)/height squared (m^2^). Different thresholds of BMI were set according to the World Health Organization protocol (<18.5, 18.5–24.9, 25–29.9, or >30 kg/m^2^). Participants’ occupational characteristics examined included work sector (construction, finance, commercial, manufacturing, public services, transportation, or other), type of occupation (administrative, manual worker, professional, salesperson, technician, teacher, or other), time the since accident in days, type of accident (commute vs. work injury), and type of injury (burn, fall, cut, attrition, firearm, blunt trauma, repetitive use, or other).

### Statistical analysis

Frequency distributions of sociodemographic and occupational characteristics were assessed using numbers and percentages (%) for categorical variables and mean ± standard deviations (SD) for continuous variables. P-values were calculated using the Chi-square test for categorical variables and the analysis of variance (ANOVA) for continuous variables. For the SF-MPG total score and subscales, median values (± interquartile range [IQR]) were calculated, since the distribution of these values was skewed. The Wilcoxon-Mann Whitney test was used to evaluate differences in medians. Cronbach’s alpha was used to assess internal consistency for PII, SF-MPG, SF-MPG sensory subscale, and SF-MPG affective subscale. For both the PII and SF-MPG, we also analyzed each item’s reliability by assessing item-total correlations and overall reliability when a specific item was deleted. Construct validity was assessed using exploratory factor analysis (EFA). Prior to conducting the EFA, we examined the data to ensure suitability for EFA; these analyses demonstrated it was appropriate to proceed with factor analyses (For PII: Kaiser’s measure of sampling adequacy (MSA) = 0.889, Bartlett’s test of sphericity p<0.001; For SF-MPG: MSA = 0.910, Bartlett’s test of sphericity p<0.001). EFAs were conducted for the PII and SF-MPG questionnaires both separately and combined. EFAs were conducted using principal component analysis with varimax rotation. Factors with eigenvalues >1 were assumed to be meaningful and retained for rotation. Rotation factor loadings of ≥ 0.4 were considered sufficient while items with factor loadings of ≥ 0.4 on more than one factor were cross-loading. Since previous studies have shown an association between gender and pain tolerance [21, 22], exploratory analyses were performed separately for men and women. All statistical analyses were performed using SPSS Statistics, Version 23.0 (IBM SPSS v23.0, Armonk, NY, USA).

## Results

Sociodemographic characteristics of the population are presented in Table 1. Participants had a mean age of 45.87 ± 13.69 years. The majority of participants were men (72.4%), between 45–54 years (25.6%), reported high school as the highest degree of education they had completed (54.7%), were married or living with a partner (61.7%) and had a BMI that classifies them as overweight (43.2%). Compared to men, women in our study were more likely to be older, more highly educated, and less likely to be married or living with a partner (p<0.001, Table 1). Characteristics of study participants stratified by age group is also presented (S1 Table).

**Table 1:** Sociodemographic characteristics of injured working adults in Chile (N = 1,975).

Characteristics	All participants (N = 1,975)		Men (N = 1,429)		Women (N = 546)		p-value
	n	%	n	%	n	%	
Age (years), mean ± SD ^a^	45.87 ± 13.69		45.07 ± 13.93		47.96 ± 12.82		**<0.001**
18–24	143	7.2	114	8.0	29	5.3	**<0.001**
25–34	350	17.7	280	19.6	70	12.8	
35–44	371	18.8	273	19.1	98	17.9	
45–54	506	25.6	353	24.7	153	28.0	
55–64	458	23.2	305	21.3	153	28.0	
65–74	130	6.6	89	6.2	41	7.5	
75–84	17	0.9	15	1.0	2	0.4	
Country of birth							
Chile	1857	94.0	1333	93.3	524	96.0	**0.024**
Other	118	6.0	96	6.7	22	22	
Belong to indigenous/native group							
No	1911	96.8	1380	96.6	531	97.3	0.487
Yes	63	3.2	48	3.4	15	2.7	
Highest degree of education							
Elementary school	319	16.2	236	16.5	83	15.2	**<0.001**
High school	1080	54.7	845	59.1	235	43.1	
College or technical training	575	29.1	348	24.4	227	41.7	
Marital status							
Married/living with a partner	1218	61.7	971	67.9	247	45.3	**<0.001**
Single	478	24.2	313	21.9	165	30.3	
Previously married ^b^	278	14.1	145	10.1	133	24.4	
Body mass index (BMI, kg/m^2^)							
<18.5	12	0.6	5	0.4	7	1.3	0.083
18.5–24.9	477	24.2	342	24.0	135	24.9	
25–29.9	850	43.2	627	44.0	223	41.1	
>30	629	32.0	452	31.7	177	32.7	

^a^ based on reported age (not DOB)

^b^ Widowed, separated or divorced. For continuous variables, P-value was calculated using the ANOVA; for categorical variables, P-value was calculated using the Chi-square test. Missing values were seen for some variables, including education (n = 1), marital status (n = 1) and BMI (n = 7).

Occupational, injury, and pain characteristics of the population are shown in Table 2. The majority of participants listed their occupation as manual workers (58.0%) and the median time since accident was 189 [IQR: 71–539] days. Nearly two-thirds of participants were injured during work (65.4%). Falls (35.4%) or blunt trauma (32.7%) were the most common causes of injury. The PII mean score was 3.84 ± 1.43 among all participants. The SF-MPG median score was 11 [IQR: 6–16]. Women were significantly more likely to report higher PII and SF-MPG scores than men (p<0.001; Table 2). Overall, those in the older age ranges (35–54 years and 55–88 years) were more likely to have higher values of the PII and SF-MPG scores versus younger participants (18–34 years) (S2 Table).

**Table 2:** Occupation, injury, and pain characteristics of injured working adults in Chile (N = 1,975).

Characteristics	All participants (N = 1,975)		Men (N = 1,429)		Women (N = 546)		p-value
	n	%	n	%	n	%	
***Occupation***							
Work sector							
Construction	217	11.0	212	14.8	5	0.9	**<0.001**
Finances	36	1.8	21	1.5	15	2.7	
Commercial	361	18.3	244	17.1	117	21.4	
Manufacturing	524	26.5	425	29.7	99	18.1	
Public services	425	21.5	218	15.3	207	37.9	
Transportation	177	9.0	169	11.8	8	1.5	
Other ^a^	235	11.9	140	9.8	95	17.4	
Type of occupation							
Administrative	264	13.4	149	10.4	115	21.1	**<0.001**
Manual worker	1145	58.0	900	63.0	245	44.9	
Professional	133	6.7	80	5.6	53	9.7	
Salesperson	53	2.7	26	1.8	27	4.9	
Technician	158	8.0	108	7.6	50	9.2	
Teacher	15	0.8	2	0.1	13	2.4	
Other ^b^	206	10.4	163	11.4	43	7.9	
***Diagnosis and injury***							
Time since accident, median [IQR] days	189 [71–539]		182 [65–538]		209.5 [88.75–548]		**0.212**
Days since accident							
Acute	593	30.0	454	31.8	139	25.5	**0.005**
Sub-acute	981	49.7	679	47.5	302	55.3	
Chronic	401	20.3	296	20.7	105	19.2	
Type of accident							
Commute	682	34.6	406	28.4	276	50.6	**<0.001**
Work injury	1291	65.4	1022	71.6	269	49.4	
Type of injury							
Burn	49	2.5	35	2.5	14	2.6	**<0.001**
Fall	700	35.4	428	30.0	272	49.8	
Cut	195	9.9	176	12.3	19	3.5	
Attrition	251	12.7	227	15.9	24	4.4	
Firearm	35	1.8	34	2.4	1	0.2	
Blunt trauma	646	32.7	469	32.8	177	32.4	
Repetitive use	7	0.4	5	0.4	2	0.4	
Other	91	4.6	54	3.8	37	6.8	
***Pain Interference Index (PII)***							
Mean score, mean ±SD (range 0–6)	3.84 ± 1.43		3.68 ± 1.43		4.26 ± 1.33		**<0.001**
Total score, mean ±SD (range 0–36)	23.06 ± 8.58		22.10 ± 8.60		25.58 ± 8.00		**<0.001**
***Pain Rating Index (SF-MPQ)***							
Total score, median ± IQR (range 0–45)	11 [6, 16]		10 [6, 15]		13 [8, 20]		**<0.001**
Sensory scale, median ± IQR (range 0–33)	9 [5, 13]		8 [5, 12]		11 [7, 15]		**<0.001**
Affective scale, median ± IQR (range 0–12)	2 [0, 3]		1 [0, 3]		3 [1, 5]		**<0.001**

Missing values were seen for some variables, including type of accident (n = 2) and type of injury (n = 1).

^a^ Includes agriculture, education, security, cleaning services, administration, food service, automotive, mining, retired, gardener, electrical engineer, maintenance, etc.

^b^ Includes chauffeur, conductors, concierge, security guard, food service, landlord, food distribution, telecommunication, machine operator, cleaning services, etc.

The Cronbach’s alpha reliability coefficient for PII total score was 0.90 and was 0.87 for SF-MPG total score (Table 3). The SF-MPG sensory and affective subscales had Cronbach’s alphas of 0.82 and 0.71 respectively (Table 3). The item-total correlations coefficients between six items of the PII and the total scores ranged from 0.66 to 0.82 (all p-values <0.001) (S3 Table). The highest item-total correlation coefficient was for item 2 ‘has your pain made it difficult for you to do activities outside work?’ (0.83) and the lowest was for item 5 ‘has your pain affected your ability to do physical activities?’ (0.66; S3 Table). For SF-MPG, the highest item-total correlation coefficient was for item 4 ‘punishing-cruel pain’ of the affective subscale (0.629) while item 10 ‘tender pain’ of the sensory subscale had the lowest correlation (0.312) (S4 Table).

**Table 3:** Reliability statistics–Cronbach’s α coefficients of reliability of the Pain Interference Index (PII) and Short Form McGill Pain Questionnaire (SF-MPQ) among a Chilean population of injured working adults (N = 1,975).

	No. of items	All participants
**Cronbach’s alpha (PII)**	6	0.900
**Cronbach’s alpha (SF-MPQ)**	15	0.870
**Cronbach’s alpha (SF-MPQ, Sensory scale)**	11	0.820
**Cronbach’s alpha (SF-MPQ, Affective scale)**	4	0.714

Abbreviations: PII, Pain Interference Index; SF-MPQ, Short Form McGill Pain Questionnaire: Pain Rating Index

The internal consistency and item-total correlations were similar for the pain scales when examined separately for men and women (S4–S9 Tables).

Exploratory factor analysis showed a one-factor solution for the PII which accounted for 66.93% of the total variance in our population (Table 4). The results of EFA for SF-MPG among Chilean adults showed a two-factor solution with Factor 1 (Sharp Pain) and Factor Two (Aching Pain). The SF-MPG sensory subscale items 5, 6, 7, 8, 11 and affective subscale items 2, 3, and 4 loaded to Factor 1; while the other items of each subscale loaded to Factor 2. The two factors explained 53.22% of the total variance (Table 5). We performed an EFA analysis that included all 17-items from both pain scales. The results showed a 3-factor solution together explaining 57.76% of total variance. All items from SF-MPG loaded on two factors “Sharp Pain” and “Aching Pain” while the rest of the times from the PII scale loaded on “Pain Interference” (S5 Table).

**Table 4:** Item-level factor loadings resulting from exploratory factor analysis of the Pain Interference Index (PII) among a Chilean population of injured working adults (N = 1,975).

Component	Factor Loadings
	Factor 1: Pain Interference
Item 1: Has your pain made it difficult for you to do work?	0.816
Item 2: Has your pain made it difficult for you to do activities outside work (leisure activities)?	0.895
Item 3: Has your pain made it difficult for you to spend time with friends?	0.825
Item 4: Has your pain affected your mood	0.829
Item 5: Has your pain affected your ability to do physical activities (like run, walk upstairs, play sports)?	0.758
Item 6: Has your pain affected your sleep?	0.779
**% of the variance**	66.93

PCA with varimax rotation. Kaiser’s Measure of Sampling Adequacy: Overall MSA = 0.889. Bartlett’s test of sphericity: p<0.001

**Table 5:** Item-level factor loadings resulting from exploratory factor analysis of the Short Form McGill Pain Questionnaire (SF-MPQ) among a Chilean population of injured working adults (N = 1,975).

Component	Factor Loadings	
	Factor 1: Sharp Pain	Factor 2: Aching Pain
**Sensory subscale**		
Item 1: Throbbing	0.064	**0.786**
Item 2: Shooting	0.365	**0.621**
Item 3: Stabbing	0.444	**0.550**
Item 4: Sharp	0.173	**0.669**
Item 5: Cramping	**0.722**	0.099
Item 6: Gnawing	**0.627**	0.278
Item 7: Hot-burning	**0.706**	0.006
Item 8: Aching	**0.719**	0.153
Item 9: Heavy	0.117	**0.712**
Item 10: Tender	-0.171	**0.752**
Item 11: Splitting	**0.739**	0.044
**Affective subscale**		
Item 1: Tiring-exhausting	0.251	**0.691**
Item 2: Sickening	**0.678**	0.125
Item 3: Fearful	**0.769**	0.120
Item 4: Punishing-cruel	**0.655**	0.332
**% of the variance**	53.22	

PCA with varimax rotation. Kaiser’s Measure of Sampling Adequacy: Overall MSA = 0.910. Bartlett’s test of sphericity: p<0.001

## Discussion

The Spanish-language versions of the PII and SF-MPG had good construct validity, concurrent validity, and internal consistency for assessing chronic pain when administered to injured working Chilean adults. The EFA results indicated that the PII was a unidimensional scale while the SF-MPG had a two-factor solution. Furthermore, the EFA analysis on the pooled items resulted in a 3-factor structure of “Sharp Pain”, “Throbbing Pain”, and “Pain Interference”.

Three previous studies have examined the psychometric properties of PII [15, 23, 24]. Martin *et al* examined the PII among 60 English-speaking patients (ages 6 to 24 years) and their parents in Sweden. The PII showed good internal consistency with a Cronbach alpha of 0.84 for the patients, and 0.94 and 0.96 for the parents (mothers and fathers, respectively) [15]. Among children and adolescents at a tertiary pain clinic in Sweden [N = 163, ages 7 to 18 years), Holmström *et al* found a one-factor solution with a Cronbach’s alpha of 0.856 for a Swedish-language version of the PII [23]. In a similar patient population of 205 Swedish adults at a tertiary pain clinic (ages 18 to 85 years), the PII showed a one-factor solution and an overall Cronbach’s alpha of 0.85 [24]. Taken together, these results are concordant with the results of our study of the Spanish-language version of the PII used among Chilean adults. Namely, like the previous studies, we found a one-factor solution and a Cronbach’s alpha reliability coefficient of 0.90 for the PII. To our knowledge, PII has not been previously validated in Spanish, but other pain scales have been translated to measure their adequacy in Spanish-speaking populations with back injuries, obtaining good assessments of chronic pain [25, 26].

Few previous studies have examined the psychometric properties of the SF-MPG. The SF-MPG was first designed and tested in French and English-speaking patient populations in Montreal, Canada [group 1: post-surgical (N = 40), obstetrical (N = 20, and musculoskeletal (N = 10); group 2: post-surgical (N = 31) and dental (N = 31)). Melzack showed there was a high correlation between the SF-MPG and the original long form-McGill Pain Index for measuring pain symptoms [18]. Wright *et al* examined 188 Canadian patients with chronic back pain and performed a confirmatory factor analysis (CFA) to test the validity of the two-factor model proposed by Melzack. Wright *et al* found a two-factor solution for the SF-MPG. However, in Wright *et al*. item 6 (gnawing) loaded as an affective pain item compared to Melzack *et al*. where item 6 loaded as a sensory item [18, 19]. The Cronbach’s alpha for the final model was 0.77 [19]. Lastly, a study of 100 female Swedish patients (50 with fibromyalgia and 50 with rheumatoid arthritis) showed a three-factor solution with factors of acute sensory, chronic sensory, and affective pain [27]. In our population of working Spanish-speaking Chilean adults, the SF-MPG loaded as two factors with both sensory and affective items loading to each of the factors. In other Spanish-speaking populations, Lázaro *et al*. evaluated the internal consistency of the long form of the MPG, confirming its psychometric validity, but no reliability studies were conducted [28]. As for the SF-MPG, we did not find applications of the Spanish version of this index, which shows the relevance of our findings to identify pain in Spanish-speaking countries.

The strengths of our study included large sample size, the use of well-trained interviewers for data collection, and our conduct of sensitivity analyses designed to explore the influence of gender on the chronic pain scales. However, our study also had some limitations. Our population of working adults at a workers’ compensation hospital may not be generalizable to other adult populations in South America, because it focuses on injured workers, excluding informal workers, those beneficiaries of the public system, and non-injured workers. Thus, future studies are needed to include these participants and evaluate if the instruments perform similarly. Additionally, we were unable to evaluate criterion validity, since there is no gold standard for measuring the impacts of chronic pain. It is also possible that translation differences of the SF-MPG may have affected the factor structure. Our EFA showed a two-factor solution for the SF-MPG. Previous studies have shown two-factor structures for English and French versions of the SF-MPG [18, 19] and a three-factor solution for the Swedish version [27].

## Conclusions

Our study was the first to evaluate the reliability, construct validity and concurrent validity of PII and SF-MPG among Spanish speaking Chilean adults. Overall, both the PII and SF-MPG have good construct validity and reliability for assessing different aspects of chronic pain in a population of injured working adults. The PII assesses the extent to which pain interferes with daily activities, while the SF-MPG measures the dimensions of the pain that participants experience. Identification of chronic pain is important for clinical diagnosis and follow-up treatments for these conditions.

## Supporting information

S1 Table Sociodemographic characteristics of injured working adults in Chile by age groups (N = 1,975). (DOCX)

S2 Table Occupation, injury, and pain characteristics of injured working adults in Chile by age groups (N = 1,975). (DOCX)

S3 Table Item characteristics, item-total correlation, alpha if item deleted of the Pain Interference Index (PII) among a Chilean population of injured working adults (N = 1,975). (DOCX)

S4 Table Item characteristics, item-total correlation, alpha if item deleted of the Short Form McGill Pain Questionnaire (SF-MPQ) among a Chilean population of injured working adults (N = 1,975). (DOCX)

S5 Table Item-level factor loadings resulting from combined exploratory factor analysis of the Pain Interference Index (PII) and Short Form McGill Pain Questionnaire (SF-MPQ) among a Chilean population of injured working adults (N = 1,975). (DOCX)

S6 Table Reliability statistics–Cronbach’s α coefficients of reliability of the Pain Interference Index (PII) and Short Form McGill Pain Questionnaire (SF-MPQ) among injured men and women in a working Chilean population (N = 546–1,429). (DOCX)

S7 Table Item characteristics, item-total correlation, alpha if item deleted of the Pain Interference Index (PII) among injured men and women in a working Chilean population (N = 546–1,429). (DOCX)

S8 Table Item characteristics, item-total correlation, alpha if item deleted of the Short Form McGill Pain Questionnaire (SF-MPQ) among injured men and women in a working Chilean population (N = 546–1,429). (DOCX)

S9 Table Item-level factor loadings resulting from exploratory factor analysis of the Pain Interference Index (PII) among injured men and women in a working Chilean population (N = 546–1,429). (DOCX)

S10 Table Item-level factor loadings resulting from exploratory factor analysis of the Short Form McGill Pain Questionnaire (SF-MPQ) among injured men and women in a working Chilean population (N = 546–1,429). (DOCX)

S11 Table Item-level factor loadings resulting from combined exploratory factor analysis of the Pain Interference Index (PII) and Short Form McGill Pain Questionnaire (SF-MPQ) among injured men in a working Chilean population (N = 546–1,429). (DOCX)

S1 Checklist Checklist of inclusivity in global research. (DOCX)
