# Supplementary figures and images for: Evaluating the performance of the Pain Interference Index and the Short Form McGill Pain Questionnaire among Chilean injured working adults
Source: PLoS One. 2022 May 19;17(5):e0268672. doi: 10.1371/journal.pone.0268672 (PMC9119477; doi:10.1371/journal.pone.0268672)

**S1 Table.** Sociodemographic characteristics of injured working adults in Chile by age groups (N = 1,975).

**
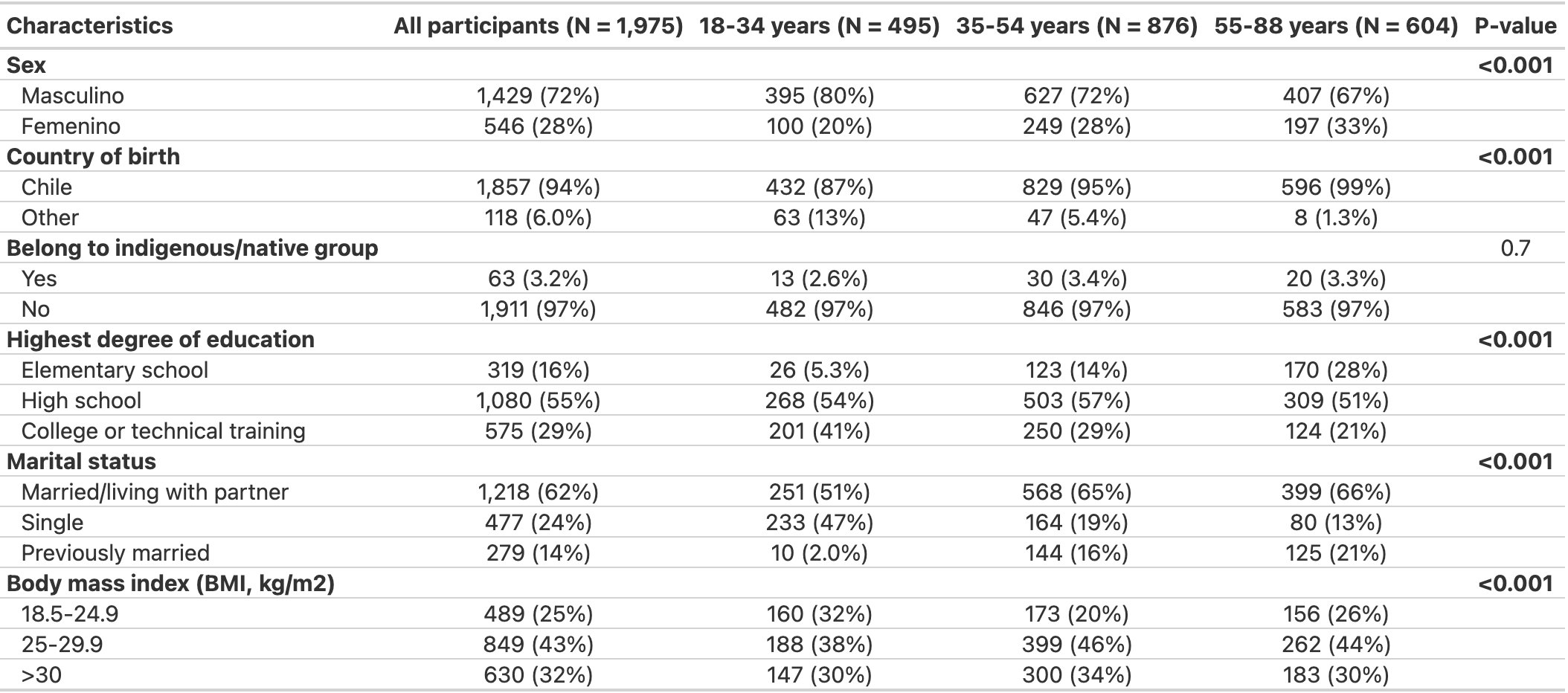
**

Supplement: S1 Table — (DOCX) [file pone.0268672.s001.docx]
