# Supplementary material for: Evaluating the performance of the Pain Interference Index and the Short Form McGill Pain Questionnaire among Chilean injured working adults
Source: PLoS One. 2022 May 19;17(5):e0268672. doi: 10.1371/journal.pone.0268672 (PMC9119477; doi:10.1371/journal.pone.0268672)
Supplement: S2 Table — (DOCX) [file pone.0268672.s002.docx]

**S2 Table.** Occupation, injury, and pain characteristics of injured working adults in Chile by age groups (N = 1,975).

**
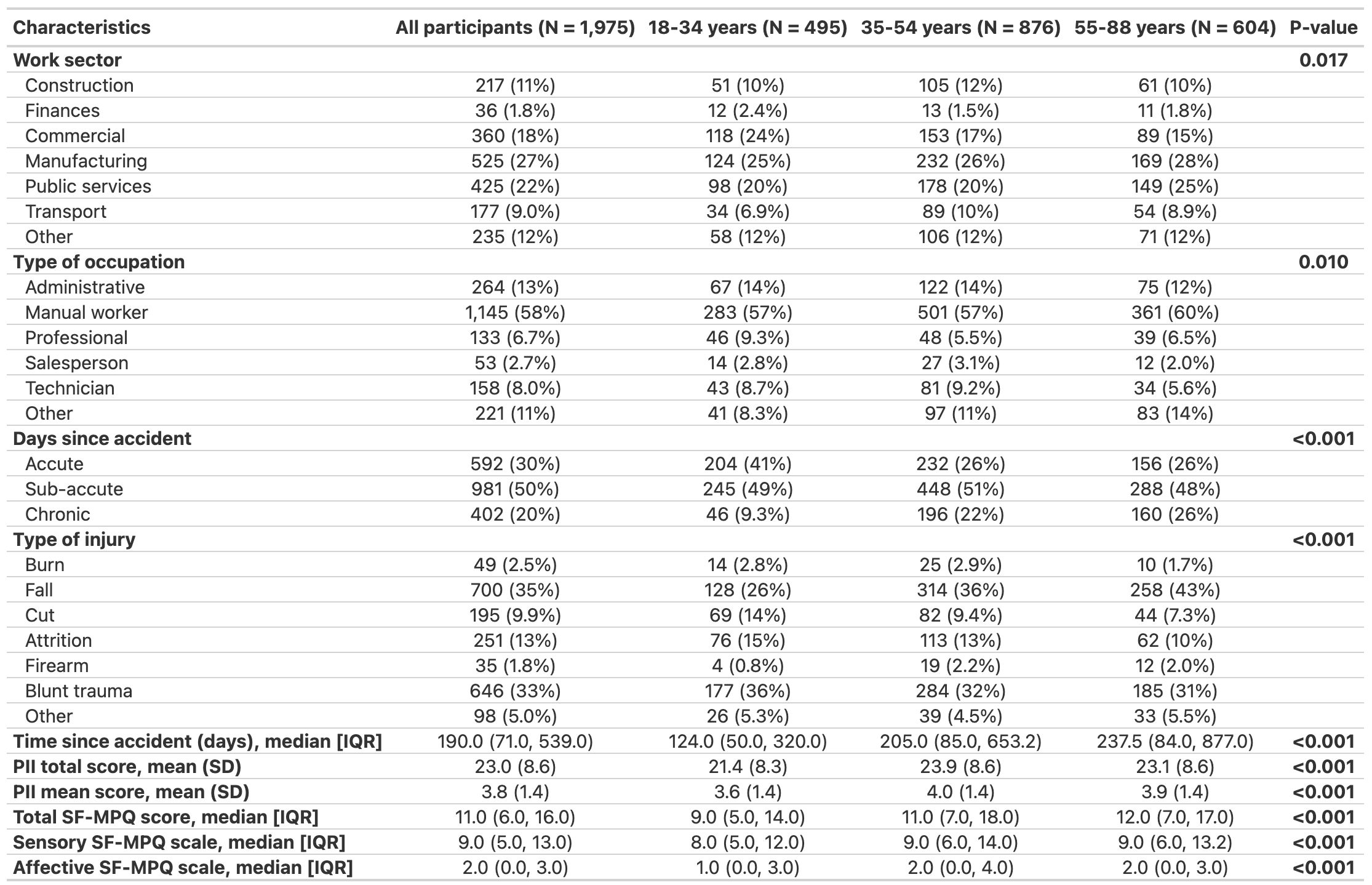
** PII, Pain Interference Index; SF-MPQ, Short Form McGill Pain Questionnaire. Work sector “other” includes teacher, agriculture, education, security, cleaning services, administration, food service, automotive, mining, retired, gardener, electrical engineer, maintenance, etc. Missing values were seen for some variables, including education (n=1), marital status (n=1) and BMI (n=7). For continuous variables, P-value was calculated using the ANOVA; for categorical variables, P-value was calculated using the Chi-square test.
