# Supplementary material for: Evaluating the performance of the Pain Interference Index and the Short Form McGill Pain Questionnaire among Chilean injured working adults
Source: PLoS One. 2022 May 19;17(5):e0268672. doi: 10.1371/journal.pone.0268672 (PMC9119477; doi:10.1371/journal.pone.0268672)
Supplement: S3 Table — (DOCX) [file pone.0268672.s003.docx]

**S3 Table.** Item characteristics, item-total correlation, alpha if item deleted of the Pain Interference Index (PII) among a Chilean population of injured working adults (N = 1,975).

| **Component** | **Range** | **Mean** | **SD** | **Corrected Item-Total Correlation** | **Alpha if Item Deleted** |
| --- | --- | --- | --- | --- | --- |
| Item 1: Has your pain made it difficult for you to do work? | [0,6] | 4.09 | 1.68 | 0.722 | 0.883 |
| Item 2: Has your pain made it difficult for you to do activities outside work (leisure activities)? | [0,6] | 3.64 | 1.67 | 0.832 | 0.867 |
| Item 3: Has your pain made it difficult for you to spend time with friends? | [0,6] | 3.25 | 1.85 | 0.733 | 0.881 |
| Item 4: Has your pain affected your mood | [0,6] | 3.83 | 1.82 | 0.743 | 0.880 |
| Item 5: Has your pain affected your ability to do physical activities (like run, walk upstairs, play sports)? | [0,6] | 4.40 | 1.67 | 0.660 | 0.892 |
| Item 6: Has your pain affected your sleep? | [0,6] | 3.85 | 1.82 | 0.684 | 0.889 |
