# Supplementary material for: Evaluating the performance of the Pain Interference Index and the Short Form McGill Pain Questionnaire among Chilean injured working adults
Source: PLoS One. 2022 May 19;17(5):e0268672. doi: 10.1371/journal.pone.0268672 (PMC9119477; doi:10.1371/journal.pone.0268672)
Supplement: S4 Table — (DOCX) [file pone.0268672.s004.docx]

**S4 Table.** Item characteristics, item-total correlation, alpha if item deleted of the Short Form McGill Pain Questionnaire (SF-MPQ) among a Chilean population of injured working adults (N = 1,975).

| **Component** | **Range** | **Mean** | **SD** | **Corrected Item-Total Correlation** | **Alpha if Item Deleted** |
| --- | --- | --- | --- | --- | --- |
| **Sensory subscale** |  |  |  |  |  |
| Item 1: Throbbing | [0,3] | 1.59 | 0.95 | 0.511 | 0.863 |
| Item 2: Shooting | [0,3] | 0.63 | 0.91 | 0.608 | 0.858 |
| Item 3: Stabbing | [0,3] | 0.64 | 0.92 | 0.617 | 0.858 |
| Item 4: Sharp | [0,3] | 1.75 | 0.97 | 0.509 | 0.863 |
| Item 5: Cramping | [0,3] | 0.64 | 1.00 | 0.513 | 0.862 |
| Item 6: Gnawing | [0,3] | 0.43 | 0.86 | 0.570 | 0.860 |
| Item 7: Hot burning | [0,3] | 0.49 | 0.91 | 0.441 | 0.866 |
| Item 8: Aching | [0,3] | 0.39 | 0.86 | 0.551 | 0.861 |
| Item 9: Heavy | [0,3] | 1.38 | 1.26 | 0.498 | 0.865 |
| Item 10: Tender | [0,3] | 1.78 | 1.15 | 0.312 | 0.874 |
| Item 11: Splitting | [0,3] | 0.44 | 0.88 | 0.490 | 0.864 |
| **Affective subscale** |  |  |  |  |  |
| Item 1: Tiring-exhausting | [0,3] | 1.29 | 1.30 | 0.579 | 0.860 |
| Item 2: Sickening | [0,3] | 0.26 | 0.72 | 0.503 | 0.864 |
| Item 3: Fearful | [0,3] | 0.40 | 0.87 | 0.562 | 0.861 |
| Item 4: Punishing-cruel | [0,3] | 0.50 | 0.98 | 0.629 | 0.857 |
