# Supplementary material for: Evaluating the performance of the Pain Interference Index and the Short Form McGill Pain Questionnaire among Chilean injured working adults
Source: PLoS One. 2022 May 19;17(5):e0268672. doi: 10.1371/journal.pone.0268672 (PMC9119477; doi:10.1371/journal.pone.0268672)
Supplement: S5 Table — (DOCX) [file pone.0268672.s005.docx]

**S5 Table.** Item-level factor loadings resulting from combined exploratory factor analysis of the Pain Interference Index (PII) and Short Form McGill Pain Questionnaire (SF-MPQ) among a Chilean population of injured working adults (N = 1,975).

| **Component** | **Factor Loadings** | | |
| --- | --- | --- | --- |
|  | **Factor 1:**  **Sharp Pain** | **Factor 2:**  **Pain Interference** | **Factor 3: Aching Pain** |
| **Pain Interference Index** |  |  |  |
| Item 1: Has your pain made it difficult for you to do work? | 0.076 | **0.745** | 0.343 |
| Item 2: Has your pain made it difficult for you to do activities outside work (leisure activities)? | 0.137 | **0.834** | 0.292 |
| Item 3: Has your pain made it difficult for you to spend time with friends? | 0.069 | **0.793** | 0.224 |
| Item 4: Has your pain affected your mood | 0.187 | **0.758** | 0.272 |
| Item 5: Has your pain affected your ability to do physical activities (like run, walk upstairs, play sports)? | 0.185 | **0.780** | -0.028 |
| Item 6: Has your pain affected your sleep? | 0.245 | **0.755** | 0.080 |
| **SF-MPQ: Sensory subscale** |  |  |  |
| Item 1: Throbbing | 0.059 | 0.187 | **0.763** |
| Item 2: Shooting | 0.382 | 0.071 | **0.624** |
| Item 3: Stabbing | 0.444 | 0.164 | **0.528** |
| Item 4: Sharp | 0.159 | 0.257 | **0.613** |
| Item 5: Cramping | **0.714** | 0.128 | 0.073 |
| Item 6: Gnawing | **0.629** | 0.083 | 0.274 |
| Item 7: Hot-burning | **0.691** | 0.157 | -0.036 |
| Item 8: Aching | **0.725** | 0.037 | 0.159 |
| Item 9: Heavy | 0.114 | 0.193 | **0.677** |
| Item 10: Tender | -0.158 | 0.070 | **0.756** |
| Item 11: Splitting | **0.736** | 0.107 | 0.015 |
| **SF-MPQ: Affective subscale** |  |  |  |
| Item 1: Tiring-exhausting | 0.251 | 0.181 | **0.661** |
| Item 2: Sickening | **0.669** | 0.117 | 0.107 |
| Item 3: Fearful | **0.755** | 0.156 | 0.090 |
| Item 4: Punishing-cruel | **0.634** | 0.226 | 0.293 |
| **% of the variance** | 57.76 | | |

PCA with varimax rotation. Kaiser's Measure of Sampling Adequacy: Overall MSA = 0.926. Bartlett's test of sphericity: p<0.001
