# Supplementary material for: Evaluating the performance of the Pain Interference Index and the Short Form McGill Pain Questionnaire among Chilean injured working adults
Source: PLoS One. 2022 May 19;17(5):e0268672. doi: 10.1371/journal.pone.0268672 (PMC9119477; doi:10.1371/journal.pone.0268672)
Supplement: S6 Table — (DOCX) [file pone.0268672.s006.docx]

**S6a Table.** Reliability statistics – Cronbach’s α coefficients of reliability of the Pain Interference Index (PII) and Short Form McGill Pain Questionnaire (SF-MPQ) among injured men in a working Chilean population (N = 1,429).

|  | **No. of items** | **All participants** |
| --- | --- | --- |
| **Cronbach’s alpha (PII)** | 6 | 0.897 |
| **Cronbach’s alpha (SF-MPQ)** | 15 | 0.865 |
| **Cronbach’s alpha (SF-MPQ, Sensory scale)** | 11 | 0.819 |
| **Cronbach’s alpha (SF-MPQ, Affective scale)** | 4 | 0.668 |

Abbreviations: PII, Pain Interference Index; SF-MPQ, Short Form McGill Pain Questionnaire: Pain Rating Index

**S6b Table.** Reliability statistics – Cronbach’s α coefficients of reliability of the Pain Interference Index (PII) and Short Form McGill Pain Questionnaire (SF-MPQ) among injured women in a working Chilean population (N = 546).

|  | **No. of items** | **All participants** |
| --- | --- | --- |
| **Cronbach’s alpha (PII)** | 6 | 0.893 |
| **Cronbach’s alpha (SF-MPQ)** | 15 | 0.869 |
| **Cronbach’s alpha (SF-MPQ, Sensory scale)** | 11 | 0.810 |
| **Cronbach’s alpha (SF-MPQ, Affective scale)** | 4 | 0.757 |

Abbreviations: PII, Pain Interference Index; SF-MPQ, Short Form McGill Pain Questionnaire: Pain Rating Index
