# Supplementary material for: Evaluating the performance of the Pain Interference Index and the Short Form McGill Pain Questionnaire among Chilean injured working adults
Source: PLoS One. 2022 May 19;17(5):e0268672. doi: 10.1371/journal.pone.0268672 (PMC9119477; doi:10.1371/journal.pone.0268672)
Supplement: S7 Table — (DOCX) [file pone.0268672.s007.docx]

**S7a Table.** Item characteristics, item-total correlation, alpha if item deleted of the Pain Interference Index (PII) among injured men in a working Chilean population (N = 1,429).

| **Component** | **Range** | **Mean** | **SD** | **Corrected Item-Total Correlation** | **Alpha if Item Deleted** |
| --- | --- | --- | --- | --- | --- |
| Item 1: Has your pain made it difficult for you to do work? | [0,6] | 3.96 | 1.71 | 0.719 | 0.880 |
| Item 2: Has your pain made it difficult for you to do activities outside work (leisure activities)? | [0,6] | 3.49 | 1.66 | 0.833 | 0.863 |
| Item 3: Has your pain made it difficult for you to spend time with friends? | [0,6] | 3.09 | 1.82 | 0.727 | 0.879 |
| Item 4: Has your pain affected your mood | [0,6] | 3.63 | 1.83 | 0.738 | 0.877 |
| Item 5: Has your pain affected your ability to do physical activities (like run, walk upstairs, play sports)? | [0,6] | 4.27 | 1.71 | 0.663 | 0.888 |
| Item 6: Has your pain affected your sleep? | [0,6] | 3.66 | 1.85 | 0.667 | 0.888 |

**S7b Table.** Item characteristics, item-total correlation, alpha if item deleted of the Pain Interference Index (PII) among injured women in a working Chilean population (N = 546).

| **Component** | **Range** | **Mean** | **SD** | **Corrected Item-Total Correlation** | **Alpha if Item Deleted** |
| --- | --- | --- | --- | --- | --- |
| Item 1: Has your pain made it difficult for you to do work? | [0,6] | 4.43 | 1.55 | 0.710 | 0.875 |
| Item 2: Has your pain made it difficult for you to do activities outside work (leisure activities)? | [0,6] | 4.05 | 1.63 | 0.813 | 0.859 |
| Item 3: Has your pain made it difficult for you to spend time with friends? | [0,6] | 3.66 | 1.85 | 0.731 | 0.873 |
| Item 4: Has your pain affected your mood | [0,6] | 4.36 | 1.68 | 0.724 | 0.873 |
| Item 5: Has your pain affected your ability to do physical activities (like run, walk upstairs, play sports)? | [0,6] | 4.75 | 1.52 | 0.620 | 0.888 |
| Item 6: Has your pain affected your sleep? | [0,6] | 4.32 | 1.67 | 0.697 | 0.877 |
