# Supplementary material for: Evaluating the performance of the Pain Interference Index and the Short Form McGill Pain Questionnaire among Chilean injured working adults
Source: PLoS One. 2022 May 19;17(5):e0268672. doi: 10.1371/journal.pone.0268672 (PMC9119477; doi:10.1371/journal.pone.0268672)
Supplement: S8 Table — (DOCX) [file pone.0268672.s008.docx]

**S8a Table.** Item characteristics, item-total correlation, alpha if item deleted of the Short Form McGill Pain Questionnaire (SF-MPQ) among injured men in a working Chilean population (N = 1,429).

| **Component** | **Range** | **Mean** | **SD** | **Corrected Item-Total Correlation** | **Alpha if Item Deleted** |
| --- | --- | --- | --- | --- | --- |
| **Sensory subscale** |  |  |  |  |  |
| Item 1: Throbbing | [0,3] | 1.53 | 0.94 | 0.523 | 0.856 |
| Item 2: Shooting | [0,3] | 0.60 | 0.90 | 0.633 | 0.850 |
| Item 3: Stabbing | [0,3] | 0.60 | 0.90 | 0.637 | 0.850 |
| Item 4: Sharp | [0,3] | 1.69 | 0.96 | 0.532 | 0.855 |
| Item 5: Cramping | [0,3] | 0.57 | 0.94 | 0.479 | 0.858 |
| Item 6: Gnawing | [0,3] | 0.38 | 0.82 | 0.564 | 0.854 |
| Item 7: Hot burning | [0,3] | 0.42 | 0.85 | 0.397 | 0.862 |
| Item 8: Aching | [0,3] | 0.34 | 0.80 | 0.533 | 0.856 |
| Item 9: Heavy | [0,3] | 1.28 | 1.24 | 0.520 | 0.857 |
| Item 10: Tender | [0,3] | 1.71 | 1.16 | 0.328 | 0.868 |
| Item 11: Splitting | [0,3] | 0.41 | 0.84 | 0.451 | 0.859 |
|  |  |  |  |  |  |
| **Affective subscale** |  |  |  |  |  |
| Item 1: Tiring-exhausting | [0,3] | 1.19 | 1.27 | 0.564 | 0.855 |
| Item 2: Sickening | [0,3] | 0.21 | 0.64 | 0.449 | 0.860 |
| Item 3: Fearful | [0,3] | 0.31 | 0.77 | 0.534 | 0.856 |
| Item 4: Punishing-cruel | [0,3] | 0.39 | 0.89 | 0.614 | 0.851 |

**S8b Table.** Item characteristics, item-total correlation, alpha if item deleted of the Short Form McGill Pain Questionnaire (SF-MPQ) among injured women in a working Chilean population (N = 546).

| **Component** | **Range** | **Mean** | **SD** | **Corrected Item-Total Correlation** | **Alpha if Item Deleted** |
| --- | --- | --- | --- | --- | --- |
| **Sensory subscale** |  |  |  |  |  |
| Item 1: Throbbing | [0,3] | 1.76 | 0.97 | 0.458 | 0.864 |
| Item 2: Shooting | [0,3] | 0.71 | 0.94 | 0.559 | 0.859 |
| Item 3: Stabbing | [0,3] | 0.73 | 0.96 | 0.577 | 0.858 |
| Item 4: Sharp | [0,3] | 1.90 | 0.98 | 0.438 | 0.865 |
| Item 5: Cramping | [0,3] | 0.83 | 1.11 | 0.552 | 0.859 |
| Item 6: Gnawing | [0,3] | 0.56 | 0.95 | 0.568 | 0.859 |
| Item 7: Hot burning | [0,3] | 0.66 | 1.04 | 0.497 | 0.862 |
| Item 8: Aching | [0,3] | 0.51 | 0.98 | 0.573 | 0.859 |
| Item 9: Heavy | [0,3] | 1.64 | 1.26 | 0.416 | 0.867 |
| Item 10: Tender | [0,3] | 1.96 | 1.12 | 0.242 | 0.875 |
| Item 11: Splitting | [0,3] | 0.52 | 0.96 | 0.566 | 0.859 |
|  |  |  |  |  |  |
| **Affective subscale** |  |  |  |  |  |
| Item 1: Tiring-exhausting | [0,3] | 1.57 | 1.32 | 0.584 | 0.858 |
| Item 2: Sickening | [0,3] | 0.41 | 0.89 | 0.575 | 0.859 |
| Item 3: Fearful | [0,3] | 0.63 | 1.05 | 0.583 | 0.858 |
| Item 4: Punishing-cruel | [0,3] | 0.79 | 1.15 | 0.628 | 0.855 |
