# Supplementary material for: Evaluating the performance of the Pain Interference Index and the Short Form McGill Pain Questionnaire among Chilean injured working adults
Source: PLoS One. 2022 May 19;17(5):e0268672. doi: 10.1371/journal.pone.0268672 (PMC9119477; doi:10.1371/journal.pone.0268672)
Supplement: S9 Table — (DOCX) [file pone.0268672.s009.docx]

**S9a Table.** Item-level factor loadings resulting from exploratory factor analysis of the Pain Interference Index (PII) among injured men in a working Chilean population (N = 1,429).

| **Component** | **Factor Loadings** |
| --- | --- |
|  | **Factor 1: Pain Interference** |
| Item 1: Has your pain made it difficult for you to do work? | 0.814 |
| Item 2: Has your pain made it difficult for you to do activities outside work (leisure activities)? | 0.897 |
| Item 3: Has your pain made it difficult for you to spend time with friends? | 0.822 |
| Item 4: Has your pain affected your mood | 0.826 |
| Item 5: Has your pain affected your ability to do physical activities (like run, walk upstairs, play sports)? | 0.761 |
| Item 6: Has your pain affected your sleep? | 0.766 |
|  |  |
| **% of the variance** | 66.53 |

PCA with varimax rotation. Kaiser's Measure of Sampling Adequacy: Overall MSA = 0.884. Bartlett's test of sphericity: p<0.001

**S9b Table.** Item-level factor loadings resulting from exploratory factor analysis of the Pain Interference Index (PII) among injured women in a working Chilean population (N = 546).

| **Component** | **Factor Loadings** |
| --- | --- |
|  | **Factor 1: Pain Interference** |
| Item 1: Has your pain made it difficult for you to do work? | 0.806 |
| Item 2: Has your pain made it difficult for you to do activities outside work (leisure activities)? | 0.883 |
| Item 3: Has your pain made it difficult for you to spend time with friends? | 0.822 |
| Item 4: Has your pain affected your mood | 0.816 |
| Item 5: Has your pain affected your ability to do physical activities (like run, walk upstairs, play sports)? | 0.726 |
| Item 6: Has your pain affected your sleep? | 0.793 |
|  |  |
| **% of the variance** | 65.46 |

PCA with varimax rotation. Kaiser's Measure of Sampling Adequacy: Overall MSA = 0.885. Bartlett's test of sphericity: p<0.001
