# Supplementary material for: Evaluating the performance of the Pain Interference Index and the Short Form McGill Pain Questionnaire among Chilean injured working adults
Source: PLoS One. 2022 May 19;17(5):e0268672. doi: 10.1371/journal.pone.0268672 (PMC9119477; doi:10.1371/journal.pone.0268672)
Supplement: S10 Table — (DOCX) [file pone.0268672.s010.docx]

**S10a Table.** Item-level factor loadings resulting from exploratory factor analysis of the Short Form McGill Pain Questionnaire (SF-MPQ) among injured men in a working Chilean population (N = 1,429).

| **Component** | **Factor Loadings** | |
| --- | --- | --- |
|  | **Factor 1: Sharp Pain** | **Factor 2: Aching Pain** |
| **Sensory subscale** |  |  |
| Item 1: Throbbing | 0.053 | **0.785** |
| Item 2: Shooting | 0.374 | **0.638** |
| Item 3: Stabbing | 0.431 | **0.586** |
| Item 4: Sharp | 0.185 | **0.670** |
| Item 5: Cramping | **0.700** | 0.098 |
| Item 6: Gnawing | **0.646** | 0.266 |
| Item 7: Hot burning | **0.678** | -0.005 |
| Item 8: Aching | **0.703** | 0.169 |
| Item 9: Heavy | 0.132 | **0.704** |
| Item 10: Tender | -0.180 | **0.754** |
| Item 11: Splitting | **0.731** | 0.024 |
|  |  |  |
| **Affective subscale** |  |  |
| Item 1: Tiring-exhausting | 0.220 | **0.687** |
| Item 2: Sickening | **0.616** | 0.136 |
| Item 3: Fearful | **0.750** | 0.130 |
| Item 4: Punishing-cruel | **0.624** | 0.361 |
|  |  |  |
| **% of the variance** | 52.09 | |

PCA with varimax rotation. Kaiser's Measure of Sampling Adequacy: Overall MSA = 0.904. Bartlett's test of sphericity: p<0.001.

**S10b Table.** Item-level factor loadings resulting from exploratory factor analysis of the Short Form McGill Pain Questionnaire (SF-MPQ) among injured women in a working Chilean population (N = 546).

| **Component** | **Factor Loadings** | |
| --- | --- | --- |
|  | **Factor 1: Sharp Pain** | **Factor 2: Aching Pain** |
| **Sensory subscale** |  |  |
| Item 1: Throbbing | 0.072 | **0.777** |
| Item 2: Shooting | 0.369 | **0.573** |
| Item 3: Stabbing | 0.486 | **0.456** |
| Item 4: Sharp | 0.140 | **0.651** |
| Item 5: Cramping | **0.752** | 0.071 |
| Item 6: Gnawing | **0.596** | 0.286 |
| Item 7: Hot burning | **0.741** | -0.005 |
| Item 8: Aching | **0.743** | 0.108 |
| Item 9: Heavy | 0.066 | **0.714** |
| Item 10: Tender | -0.183 | **0.738** |
| Item 11: Splitting | **0.760** | 0.074 |
|  |  |  |
| **Affective subscale** |  |  |
| Item 1: Tiring-exhausting | 0.292 | **0.696** |
| Item 2: Sickening | **0.752** | 0.095 |
| Item 3: Fearful | **0.790** | 0.067 |
| Item 4: Punishing-cruel | **0.692** | 0.254 |
|  |  |  |
| **% of the variance** | 53.81 | |

PCA with varimax rotation. Kaiser's Measure of Sampling Adequacy: Overall MSA = 0.897. Bartlett's test of sphericity: p<0.001.
