# Supplementary material for: Evaluating the performance of the Pain Interference Index and the Short Form McGill Pain Questionnaire among Chilean injured working adults
Source: PLoS One. 2022 May 19;17(5):e0268672. doi: 10.1371/journal.pone.0268672 (PMC9119477; doi:10.1371/journal.pone.0268672)
Supplement: S11 Table — (DOCX) [file pone.0268672.s011.docx]

**S11a Table.** Item-level factor loadings resulting from combined exploratory factor analysis of the Pain Interference Index (PII) and Short Form McGill Pain Questionnaire (SF-MPQ) among injured men in a working Chilean population (N = 1,429).

| **Component** | **Factor Loadings** | | |
| --- | --- | --- | --- |
|  | **Factor 1: Sharp Pain** | **Factor 2:**  **Pain Interference** | **Factor 3:**  **Aching Pain** |
| **Pain Interference Index** |  |  |  |
| Item 1: Has your pain made it difficult for you to do work? | 0.078 | **0.740** | 0.350 |
| Item 2: Has your pain made it difficult for you to do activities outside work (leisure activities)? | 0.135 | **0.833** | 0.299 |
| Item 3: Has your pain made it difficult for you to spend time with friends? | 0.065 | **0.789** | 0.225 |
| Item 4: Has your pain affected your mood | 0.158 | **0.760** | 0.271 |
| Item 5: Has your pain affected your ability to do physical activities (like run, walk upstairs, play sports)? | 0.212 | **0.782** | -0.038 |
| Item 6: Has your pain affected your sleep? | 0.229 | **0.747** | 0.079 |
| **SF-MPQ: Sensory subscale** |  |  |  |
| Item 1: Throbbing | 0.048 | 0.196 | **0.757** |
| Item 2: Shooting | 0.388 | 0.091 | **0.639** |
| Item 3: Stabbing | 0.433 | 0.164 | **0.563** |
| Item 4: Sharp | 0.171 | 0.268 | **0.610** |
| Item 5: Cramping | **0.689** | 0.139 | 0.067 |
| Item 6: Gnawing | **0.646** | 0.087 | 0.263 |
| Item 7: Hot-burning | **0.660** | 0.169 | -0.054 |
| Item 8: Aching | **0.708** | 0.044 | 0.172 |
| Item 9: Heavy | 0.131 | 0.182 | **0.672** |
| Item 10: Tender | -0.168 | 0.076 | **0.756** |
| Item 11: Splitting | **0.725** | 0.122 | -0.010 |
| **SF-MPQ: Affective subscale** |  |  |  |
| Item 1: Tiring-exhausting | 0.224 | 0.153 | **0.664** |
| Item 2: Sickening | **0.611** | 0.089 | 0.127 |
| Item 3: Fearful | **0.742** | 0.119 | 0.112 |
| Item 4: Punishing-cruel | **0.607** | 0.204 | 0.329 |
|  |  |  |  |
| **% of the variance** | 56.85 | | |

PCA with varimax rotation. Kaiser's Measure of Sampling Adequacy: Overall MSA = 0.920. Bartlett's test of sphericity: p<0.001.

**S11b Table.** Item-level factor loadings resulting from combined exploratory factor analysis of the Pain Interference Index (PII) and Short Form McGill Pain Questionnaire (SF-MPQ) among injured women in a working Chilean population (N = 546).

| **Component** | **Factor Loadings** | | |
| --- | --- | --- | --- |
|  | **Factor 1:**  **Sharp Pain** | **Factor 2:**  **Pain Interference** | **Factor 3:**  **Aching Pain** |
| **Pain Interference Index** |  |  |  |
| Item 1: Has your pain made it difficult for you to do work? | 0.056 | **0.750** | 0.310 |
| Item 2: Has your pain made it difficult for you to do activities outside work (leisure activities)? | 0.121 | **0.836** | 0.251 |
| Item 3: Has your pain made it difficult for you to spend time with friends? | 0.051 | **0.801** | 0.203 |
| Item 4: Has your pain affected your mood | 0.226 | **0.746** | 0.249 |
| Item 5: Has your pain affected your ability to do physical activities (like run, walk upstairs, play sports)? | 0.112 | **0.753** | -0.035 |
| Item 6: Has your pain affected your sleep? | 0.264 | **0.770** | 0.036 |
| **SF-MPQ: Sensory subscale** |  |  |  |
| Item 1: Throbbing | 0.072 | 0.149 | **0.770** |
| Item 2: Shooting | 0.394 | 0.027 | **0.577** |
| Item 3: Stabbing | **0.481** | 0.178 | **0.428** |
| Item 4: Sharp | 0.132 | 0.213 | **0.611** |
| Item 5: Cramping | **0.751** | 0.083 | 0.055 |
| Item 6: Gnawing | **0.603** | 0.064 | 0.279 |
| Item 7: Hot-burning | **0.731** | 0.115 | -0.033 |
| Item 8: Aching | **0.750** | 0.014 | 0.117 |
| Item 9: Heavy | 0.064 | 0.187 | **0.679** |
| Item 10: Tender | -0.160 | 0.022 | **0.752** |
| Item 11: Splitting | **0.762** | 0.081 | 0.052 |
| **SF-MPQ: Affective subscale** |  |  |  |
| Item 1: Tiring-exhausting | 0.286 | 0.230 | **0.650** |
| Item 2: Sickening | **0.738** | 0.152 | 0.065 |
| Item 3: Fearful | **0.768** | 0.196 | 0.021 |
| Item 4: Punishing-cruel | **0.669** | 0.239 | 0.207 |
|  |  |  |  |
| **% of the variance** | 57.91 | | |

PCA with varimax rotation. Kaiser's Measure of Sampling Adequacy: Overall MSA = 0.909. Bartlett's test of sphericity: p<0.001
